# Convolutional Neural Networks for Mechanistic Driver Detection in Atrial Fibrillation

**DOI:** 10.3390/ijms23084216

**Published:** 2022-04-11

**Authors:** Gonzalo Ricardo Ríos-Muñoz, Francisco Fernández-Avilés, Ángel Arenal

**Affiliations:** 1 Department of Cardiology, Instituto de Investigación Sanitaria Gregorio Marañón (IiSGM), Hospital General Universitario Gregorio Marañón, 28007 Madrid, Spain; francisco.fernandezaviles@salud.madrid.org (F.F.-A.); arenal@secardiologia.es (Á.A.); 2 Center for Biomedical Research in Cardiovascular Disease Network (CIBERCV), 28029 Madrid, Spain; 3 Departamento de Bioingeniería e Ingeniería Aeroespacial, Universidad Carlos III de Madrid, 28911 Madrid, Spain; 4 Facultad de Medicina, Universidad Complutense de Madrid, 28040 Madrid, Spain

**Keywords:** atrial fibrillation, artificial intelligence, rotors, arrhythmias, cardiology, machine learning

## Abstract

The maintaining and initiating mechanisms of atrial fibrillation (AF) remain controversial. Deep learning is emerging as a powerful tool to better understand AF and improve its treatment, which remains suboptimal. This paper aims to provide a solution to automatically identify rotational activity drivers in endocardial electrograms (EGMs) with convolutional recurrent neural networks (CRNNs). The CRNN model was compared with two other state-of-the-art methods (SimpleCNN and attention-based time-incremental convolutional neural network (ATI-CNN)) for different input signals (unipolar EGMs, bipolar EGMs, and unipolar local activation times), sampling frequencies, and signal lengths. The proposed CRNN obtained a detection score based on the Matthews correlation coefficient of 0.680, an ATI-CNN score of 0.401, and a SimpleCNN score of 0.118, with bipolar EGMs as input signals exhibiting better overall performance. In terms of signal length and sampling frequency, no significant differences were found. The proposed architecture opens the way for new ablation strategies and driver detection methods to better understand the AF problem and its treatment.

## 1. Introduction

Deep learning (DL) models are more than a trend and they are becoming practical tools in everyday life, with unprecedented performance in image classification problems, product recommendation, DNA sequence analysis, cellular electron transport, and medical diagnosis applications [1,2,3,4,5]. In the latter, DL models have recently debuted in the cardiology field to tackle problems such as arrhythmia type classification [6,7,8,9], heart rate variability [10], computed tomography (CT), magnetic resonance imaging (MRI) 3D ventricular-atrial segmentation [11], diagnosis of arrhythmias in wearable devices [12], guidance in irregular heart electrical activity, and more specifically atrial fibrillation (AF) electroanatomical mapping [13,14,15].

AF is a major problem in our society, with an estimated prevalence of 2–4% in adults [16]. The suboptimal treatment of this arrhythmia and the ongoing debate on its initiating and maintenance mechanisms, i.e., multiple wavelet propagation [17], ectopic beats [18], and rotational activity drivers [19], make AF a major challenge in modern medicine. The AF complexity calls for a holistic, multisided, and multidisciplinary approach involving the joint work of physicians, scientists, and engineers. This partnership could improve the success of AF interventions and therefore reduce AF incidence and risk [20].

**Figure 1:**
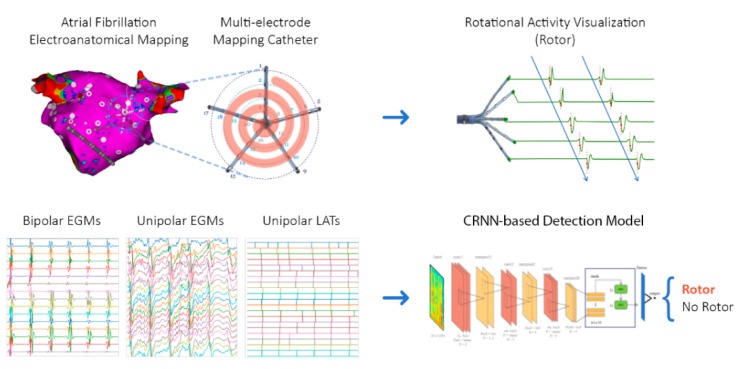
CNN-based rotational activity detection workflow. (**Top**) Endocardial EGMs registered with a multi-electrode catheter exhibiting a rotational activation (rotor) staircase pattern. Signals were obtained from a 3D electroanatomical mapping system and multi-electrode catheters. (**Bottom**) Bipolar EGMs, unipolar EGMs, and unipolar LATs are used as input signals and are fed to the proposed CRNN-based model to train and detect the presence of rotational activity in AF endocardial signals. EGMs, electrograms; LATs, local activation times; CRNN, convolutional recurrent neural network.

However, the initiating and maintenance mechanisms behind AF have generated debate and controversy. Recent technological advances allow the direct examination of the endocardial atrial tissue with multi-electrode catheters and 3D electroanatomical navigation systems. This paper aims to provide a solution to identify rotational activity drivers (rotors) using convolutional neural network (CNN)-based models from endocardial electrograms (EGMs) recorded in direct contact with the arrhythmogenic tissue. These so-called “drivers” are spatiotemporal self-reentrant electrical wavefronts that appear and propagate in the atrial tissue and may play an active role in the maintenance of AF. Multi-electrode catheters allow the visualization of the rotational activity when the signals are displayed in a ring order fashion with respect to the position and layout of the mapping catheter (Figure 1). In the presence of a rotor, bipolar EGMs exhibit a staircase activation pattern that expands the complete activation cycle and merges with the next one if repetitive rotations occur. Present signal processing algorithms that tackle the rotor detection problem use phase transformation of the EGMs [21], unipolar EGM local activation times (LATs) [22,23], causality EGM relationships [24], or even directed graphs [25] to describe this abnormal cardiac electrical propagation.

These techniques for AF driver assessment employ endocardial EGMs from multi-electrode catheters placed inside the atrial chambers. They apply traditional signal processing methods, e.g., filtering, threshold-based detectors, phase domain analysis, or fast Fourier transform [21,22,26]. Although robust, these methods require heavy pre-processing and post-processing steps that may distort the signals, lack specificity [27], and in most cases, introduce computationally expensive times to retrieve the results. DL methods, specifically CNNs, allow inner processing of the signals into feature vector representations that identify important aspects of the signals, and once they are trained, their direct application to new data can be applied in real-time signals from a patient [28], overcoming the previously mentioned limitations of traditional methods.

In this paper, we present a novel methodology to assess the presence of rotors using DL models. We propose a new network architecture based on CNNs and recurrent layers and compare its performance against two state-of-the-art models employed in electrocardiogram (ECG) classification. The rest of the paper is organized as follows. In Section 2, we present the results of three models applied to an AF EGM database for rotational activity detection. Section 3 provides a discussion of our main findings. Section 4 details the methodology, experimental settings, DL model architecture, and implementation. Finally, Section 5 summarizes the conclusions of the paper.

## 2. Results

### 2.1. Overview

We evaluated the performance of different CNN-based models to detect electrical rotational activity presence in AF endocardial EGM recordings. Figure 1 summarizes the workflow of the study and the final proposed network architecture. The first model is a simple network consisting of three dense layers with binary (sigmoid) output. The second model mimics a network architecture employed to classify ECG recordings that integrates CNN blocks and long short-term memory (LSTM) layers. The third one, our proposed model, implements CNN blocks and gated recurrent unit (GRU) layers. The detailed architecture of the models is described in the Methods section. We evaluated the performance for the two main signal acquisition configurations employed in clinical practice, i.e., unipolar and bipolar EGMs, and also for the LATs of the unipolar EGMs. Additionally, we studied the pre-processing effect when we applied increasing sub-sampling rates and different signal lengths to the input data.

### 2.2. Patient Characteristics

We retrospectively analyzed a cohort of 75 persistent AF patients (56 men, 19 women) who underwent ultra-high-density mapping during stable AF, i.e., more than 5000 points per electroanatomical map, and point-by-point pulmonary vein isolation with an electroanatomical mapping system (CARTO3 V7, Biosense Webster, Diamond Bar, CA, USA). Table 1 lists the baseline characteristics of the AF population in the study. After a first screening, patients with no rotational activity were discarded to avoid data imbalance, leaving a total of 48 patients. Patients were split into training and test sets according to a 90% training, and 10% test division (43 and 5 patients, respectively). Patients were assigned to the groups in chronological order. The first consecutive patients were assigned to the training group and the last patients to the test group.

### 2.3. DL Model Performance

We compared the performance of the three models, namely SimpleCNN, attention-based time-incremental convolutional neural network (ATI-CNN), and CRNN, for three different types of input signals: unipolar EGMs, bipolar EGMs, and unipolar LATs. Additional finer analysis was conducted regarding the sampling frequency of the data for 500, 250, and 100 Hz rates, and also varying the temporal length of the signals for 500 ms and 2500 ms segments. After training and validating the models with 34,820 and 4760 signals respectively, we tested them with 5080 new signals from different AF patients. Table 2 shows the performance results for all the parameter combinations. The best metrics for each of the models are highlighted to facilitate comparison.

**Figure 2:**
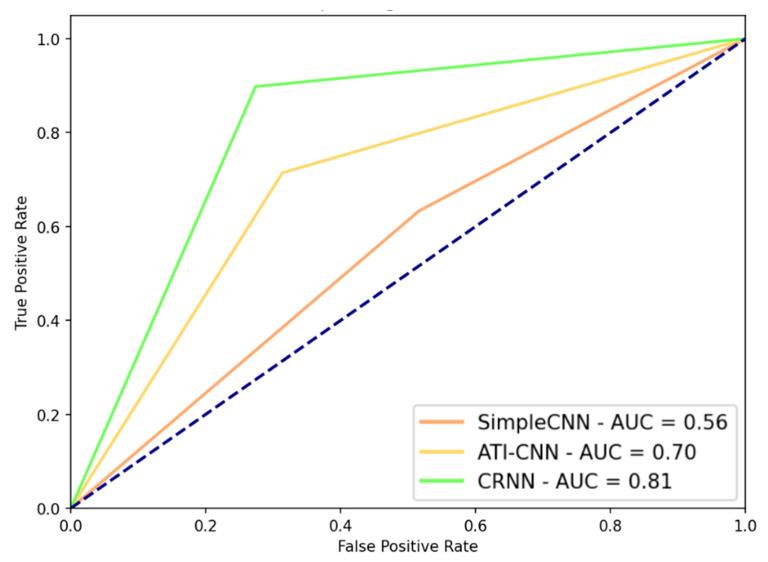
Receiver operating characteristic (ROC) curves for the three models. AUC, area under the curve.

In Figure 2, we show the receiver operating curve (ROC) for the best performance of each model. Similarly to Table 2, the CRNN model achieved the best area under the curve (AUC) = 0.81, followed by the ATI-CNN-AUC = 0.70, and SimpleCNN-AUC = 0.56.

In terms of the Matthews correlation coefficient (MCC) metric, the CRNN model achieved the best performance with MCC = 0.680 for bipolar EGMs as input signals, a sampling frequency of 500 Hz, and a signal segment length of 2500 ms. The ATI-CNN achieved MCC = 0.401 for the unipolar LATs, with a segment size of 500 ms and a sampling frequency of 100 Hz. The best result for the SimpleCNN approach was 0.118 for the unipolar LATs input, with a sampling frequency of 500 Hz and signal length of 500 ms.

Using the DL model, the SimpleCNN, ATI-CNN, and CRNN models had an average MCC performance of 0.017 ± 0.034, 0.242 ± 0.069, and 0.298 ± 0.130, respectively, for all the tested parameter combinations. The general performance of all the simulations for the three models after a one-Way ANOVA and pairwise comparison showed significant differences (*p*-value < 0.0001). Pairwise comparison between SimpleCNN and ATI-CNN and between SimpleCNN and CRNN was also significant (*p* < 0.0001). Overall CRNN performance was higher than ATI-CNN (*p* = 0.1604).

Analysis of the signal type for all the combinations showed no significant differences (*p* = 0.8449). The average performance obtained was 0.199 ± 0.174, 0.169 ± 0.124, and 0.18.14 for bipolar EGMs, unipolar EGMs, and unipolar LATs, respectively. Pairwise comparisons between signal types were not significant either.

The different sampling frequency values obtained an MCC score of 0.184 ± 0.137, 0.171 ± 0.125, and 0.199 ± 0.1956 (*p* = 0.2373) for 100 Hz, 250 Hz, and 500 Hz rates, respectively. Pairwise comparison between groups 100 Hz–250 Hz, 100 Hz–500 Hz, and 250 Hz–500 Hz provided p-values of 0.969, 0.952, and 0.8533, respectively.

Finally, the MCC for the temporal length of the input signal obtained for 500 ms and 2500 ms was 0.207 ± 0.142 and 0.161 ± 0.161, respectively (*p* = 0.2373).

## 3. Discussion

In this study, we propose the first CNN-based model for rotational activity detection in AF endocardial EGMs. The model was trained with real patient data and was tested with bipolar EGMs, unipolar EGMs, and unipolar LATs to compare their performance. In addition, we analyzed the effects of down-sampling and signal length on the detection capabilities of the proposed CNN-based models. Our proposed model was compared to two different DL models used in ECG classification. The main findings of the study were as follows: first, we highlighted the importance of bipolar EGMs for identifying rotational activity as opposed to traditional rotor assessment algorithms that focus on unipolar signals. Second, we also demonstrated their better performance versus unipolar LATs and EGMs when training DL networks. Finally, we opened the way for upcoming research on AF driver detection with leading-edge techniques, with the aim of better understanding the AF problem and its treatment.

### 3.1. Artificial Intelligence in Cardiology

Recently, DL-based methods have emerged as a powerful instrument to outperform current state-of-the-art methods and algorithms in large-scale cardiology databases. Current programming libraries, e.g., Keras or PyTorch [29,30] in Python, provide the user with friendly assets to implement neural networks; however, their real potential emerges after fine-tuning the parameters and optimizing the network design. This is the case for successful studies in the literature that employ different cardiac-related data, e.g., CT-MRI images, ECGs or EGMs, antiarrhythmic drug effects, or clinical screening [6,15,31]. The DL potential managed to provide promising results and a significant performance boost.

Given the promising results of these studies, we applied these methods and concepts to cardiac arrhythmias, and more specifically, the AF problem whose treatment remains suboptimal. The results obtained by our method are comparable with previously published works in the literature for driver detection using EGMs [32], non-invasive ECG leads [33], or optical mapping recordings [14]. Phase singularity detection by Li et al. [32] with multi-electrode catheters and virtual (in silico) EGM signals reported F-scores between 0.54 and 0.83. Their approach was based on traditional signal processing and the phase component of the signals. They concluded that AF driver identification is dependent on the phase singularity detection algorithms and their parameters, and proposed a workflow to operate with optimal parameters to facilitate the comparisons in forthcoming rotor-guided ablation. The non-invasive approach by Jones et al. [33] to determine the number of stable rotors in AF using ECG signals reported an AUC = 0.72. They applied a signal processing method, i.e., phase lock, that quantifies the spatial repeatability of fibrillatory waves in the ECG over time. Similarly, machine learning applied to optical mapping recordings by Zolotarev et al. [14] reported an accuracy of 86% for the classification of EGM recordings as an AF driver or nondriver.

These studies imply the need to better understand the mechanisms and the onset of the fibrillatory state and also to establish common metrics to compare studies that employ different technologies, signals, or algorithms. Therefore, a thorough examination of the atrial chambers is required during electroanatomical ablation procedures. In this sense the mapping time is key and is the main limitation of these studies. Mapping the atria with multi-electrode catheters requires time and expertise and can increase the overall clinical procedure, which may endanger the patient. To achieve a better trade-off between procedure time and substrate investigation, we improved the response time to obtain rotational activity detection of traditional signal processing methods with the proposed DL CRNN model. Our solution avoids heavy pre- and post-processing, minimizing the computational times. 

In addition, the model is easy to implement in most of the present programming languages and does not require specialized hardware solutions after the training process is performed. This grants future compatibility with current electrophysiology workstations, 3D electroanatomical mapping systems, or even in silico simulation platforms.

### 3.2. AF Signals

Unipolar and bipolar EGMs are the two main types of signals that electrophysiologists use to diagnose and treat cardiac arrhythmias. In the case of rotational activity, both play an active role in the detection and visualization of reentrant propagation patterns. Most of the recent commercial systems that assess rotor activity employ unipolar EGMs [22,34], which are known for providing local information about the electrical activity in the vicinity of the electrode that can accurately define the LAT of the atrial tissue. Unipolar LATs correspond to the point of maximum negative slope of the signal; however, this configuration suffers from far-field activity introduced by the ventricular activity. On the other hand, bipolar EGMs cancel this undesired noise source at the same time that they provide better readings of the local activity, but their amplitude is sensitive to the wavefront orientation and prevents the accurate identification of LATs.

Even though the rotor annotations of our database were obtained from unipolar LAT analysis, we decided to train our model with raw bipolar and unipolar EGMs, since DL networks may process the signals differently to the human brain and extract hidden features that experts may overlook or ignore. For the CRNN model, the bipolar set reported better results than the unipolar one, even when compared to the proper unipolar LATs employed to obtain the true labels. Our model correctly predicted 80.04% of the event locations; ATI-CNN performed well, with a 70.03% accuracy, and SimpleCNN reported 57.9% accuracy, not capturing the temporal dependence of the signals. One of the reasons that may explain these results is the higher signal-to-noise ratio that bipolar signals have, which probably enhances the detection of the staircase activation sequence compared to the unipolar configuration.

The type of convolution may also contribute to the better performance of the CRNN model. In our model, rectangular kernels boosted the performance of our preliminary versions with squared kernels. This enabled the network to capture longer temporal dependencies within the extracted CNN features in deeper layers, yielding satisfactory results. Hence, temporal relationships between the EGM signals are crucial to learn and correctly identify rotors from the rest of the AF propagation patterns. 

In addition, we hypothesized that the sampling frequency and the input size of the signals might have an impact on the DL learning process. However, the results in Table 2 showed that reducing the sampling frequency or the signal length did not provide a significant impact on increasing the accuracy of the learning process. The best-trained models for the CRNN and the ATI-CNN differ in sampling rates, 500 Hz and 100 Hz, respectively, which is likely related to the input signal used: bipolar EGMs for the CRNN and unipolar LATs for the ATI-CNN. The simple binary representation of the unipolar LAT signals may explain that lower sampling rates can also capture the important features and detect rotors compared to the higher variability of the bipolar EGM voltage values. They may require a higher number of samples to capture important rotor-related features. In this regard, we cannot conclude if the size or the sampling rate of the input signals is a crucial parameter to be taken into account when a DL network is designed.

### 3.3. Rotational Activity Detection Analysis

We analyzed the false positives (FPs) obtained with the CRNN model to better understand how the CRNN model worked and what the model missed. Several bipolar signals were inspected by the authors and one external cardiologist who was blinded to the detection outcome. All of them agreed that the model performance was good and that many of the FPs were in fact rotors that were misdetected in the bipolar EGMs by the CartoFinder system, which is known to provide a lack of specificity [35]. 

**Figure 3:**
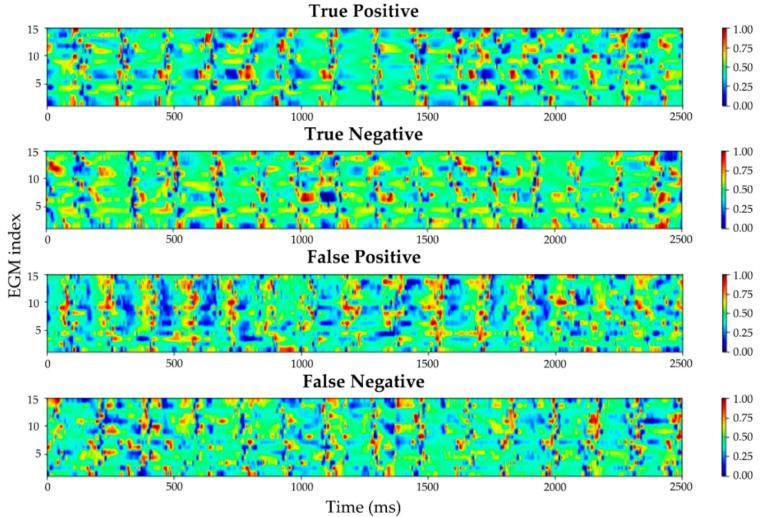
CRNN classification example. From top to bottom: the first signal shows a true positive example that exhibits a rotational activity in the last third of the temporal axis; the second signal shows a true negative classification (no rotor); the third signal shows a false positive; the fourth signal shows a false negative example of a missed rotor detection. CRNN, convolutional recurrent neural network; EGM, electrogram.

**Figure 4:**
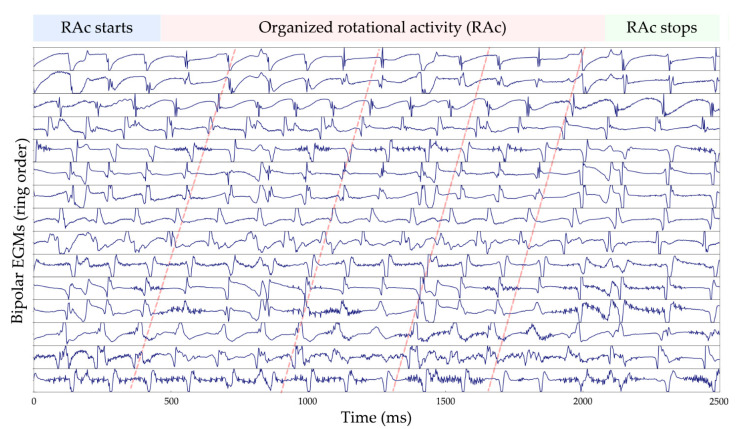
Example of an undetected rotor with CartoFinder in bipolar EGMs. The CRNN labeled this signal as a false positive, which later, after an expert inspection, was reclassified as a correctly detected rotational activity. CRNN, convolutional recurrent neural network; EGMs, electrograms; RAc, rotational activity.

As we can see in Figure 3 and Figure 4, these results validate our assumption that our model performed better than the 80.0% accuracy score in Table 2. Most of the FPs coincide with fractionation of the bipolar EGMs, which is a known surrogate characteristic of rotational activity [36]; we believe this is the main explanation for the specificity problem in the CRNN network when dealing with bipolar EGM inputs. Another explanation for this behavior can be attributed to the way we chose the “gold standard” for our database. Since DL methods usually require datasets of thousands of samples to achieve a good generalization of the problem at stake, we resorted to CartoFinder, as it could provide thousands of labeled data; this is a trusted tool to detect rotational activity, but it cannot be considered a real “gold standard”, since noise and signal artifacts may affect the EGM acquisitions. To achieve further validation of the CartoFinder method, high-resolution optical mapping videos [37] or in silico computer calculations should be employed to further determine the agreement between CartoFinder and rotational activity. Even when facing this potential limitation, our model exhibited great results and was capable of revealing new rotational activity present in bipolar EGMs that was missed by the CartoFinder module.

### 3.4. Clinical Implications of Rotational Activity

AF treatment relies on pulmonary vein isolation ablation procedures, antiarrhythmic drug administration, electrical cardioversion, or a combination of these to restore sinus rhythm. Unfortunately, they fail to optimally reduce tachycardia recurrence rates, with 50–70% recurrence after electrical cardioversion and antiarrhythmic drugs [38,39], and 20–45% after ablation procedures [38].

The impact of AF drivers like rotational activity in the management of AF is still being investigated. New mapping technologies favored in new studies evidenced solid proof of the role of rotors as a part of the AF electrophysiological substrate [40]. Clinical studies such as the FIRM trial [40,41], AFACART [42], CartoFinder driven ablation [43,44], UNCOVER AF trial [45], or the RADAR trial [46] showed initial promising results. However, there is still an open debate whether rotors are crucial to maintaining AF or if they are just bystander activations due to the collision of multiple wavefronts [47]. Recent studies have provided different outcomes; most of them were single-center studies, and they differed in the technologies employed. We believe that more extensive multicentric studies are needed to either accept or discard rotors as key components responsible for AF maintenance. In this regard, the new guidelines and studies for the management of AF open the door to new ablation strategies beyond pulmonary vein isolation [48,49].

### 3.5. Study Limitations

There are several limitations in the study. The first limitation is the absence of a gold standard database for rotor detection such as the ones employed in other fields, e.g., MNIST, ImageNet, or Labelme labeled image datasets in computer vision. For this reason, we resorted to the CartoFinder (Biosense Webster, Diamond Bar, CA, USA) module as the ground truth for our experiments. This is a single-center study, and the number of included patients could be improved. However, we consider it to be a representative number, since the CartoFinder module is a relatively new technology in the electrophysiology field and other AF-related studies with a moderate number of patients and imbalanced data have reported promising results using CNNs [7,8,9]. What is more, the algorithm requires the splines of the catheter to be correctly deployed, which in particular areas of the left atrium (LA) becomes a challenge due to its restricted accessibility and stability during the signal acquisition. In this line, surrogate indices for rotational activity detection should be further studied. Models were trained for the PentaRay catheter layout, including 20 unipolar or 15 bipolar EGMs. In the event of using a new mapping catheter model with a different number of electrodes or layout, models should be adapted and trained to the new topology and distribution of the electrodes.

## 4. Materials and Methods

In Figure 1, we visually summarize the workflow of the proposed CNN-based models to detect rotational activity in cardiac EGMs recorded with a multi-electrode catheter.

### 4.1. Patient Cohort

A total of 76 persistent AF patients were referred for their first radiofrequency ablation procedure at the Hospital General Universitario Gregorio Marañón (see Table 1). In 27 patients, no rotational activity events were found. Therefore, we included the remaining patients in the training/validation and test sets in a 90:10% split, respectively. The study was approved by the local ethics committee of Hospital General Universitario Gregorio Marañón.

### 4.2. Signal Database

Ultra-high-density endocardial maps (>5000 points) were performed with a 20-pole multi-electrode PentaRay catheter and CARTO3 V7 electroanatomical mapping system (Biosense Webster, Diamond Bar, CA, USA). Signals were bandpass filtered: bipolar EGMs at 30–240 Hz, and unipolar EGMs at 0.1–100 Hz. A 50 Hz notch was applied at a sampling frequency of 1 kHz. Signals were acquired sequentially with no pre-established order in the atrial chamber until LA mapping was completed. The setting of the electroanatomical mapping system to integrate a point in the 3D map was a point density < 2 mm, position stability of 2 mm, and the tissue proximity index enabled.

Before performing the pulmonary vein isolation ablation, the CartoFinder module for rotational detection assessment was employed (Biosense Webster, Diamond Bar, CA, USA). The module required the catheter to be correctly deployed and to remain stable for 30 s. After the acquisition, the module detected the LATs of each unipolar EGM as the time instant of the maximum negative slope of the signals. The algorithm arranged the electrode splines by rings and detected rotational activity if the LATs of the concentric circles expanded for more than 50% of the dominant cycle of the EGMs and if there were more than 2 consecutive events. The detected rotational activity should exhibit a staircase activation sequence (see Figure 1), with the upward or downward direction related to a clockwise or counter-clockwise gyre of the rotation. A total of 2792 CartoFinder acquisitions were exported for training and validating the models.

### 4.3. Signal Pre-Processing

The dataset consisted of a total of 2792 CartoFinder acquisitions. The original dataset suffered from imbalanced learning; the class imbalance ratio between rotational activity and no rotational activity was 4:100, with 27 patients (36.0%) not presenting rotor events. To compensate for this problem, we discarded the patients with no rotational activity and rebalanced the dataset by selecting as many non-rotor and rotor signals from each patient in a 1:1 ratio, which left a total of 4466 CartoFinder acquisitions for training and testing the models.

To reduce disk space, the signals were down-sampled to 500 Hz and reshaped into 15 × 15,000 and 20 × 15,000 matrices for the bipolar and unipolar EGMs, respectively. At this point, offline data augmentation was applied to increase the size of the dataset.

Data augmentation consisted of a temporal sliding window, horizontal and vertical flips, and electrode-ring order arrangement. We set a 50% sliding window overlap, and the window length was tested for two different lengths, i.e., 500 and 2500 ms. Signals were arranged by rings, and the electrode order was modified starting at spline 1 for the first augmentation and starting at spline 5 for the 5th and last permutation, which provided a 5-fold augmentation. The final unipolar and bipolar datasets contained a total of 39,580 training and 5080 test samples (4760 training samples were set aside for validation).

All signals were normalized to be in the 0–1 range. Upper and lower normalization limits were obtained from the 2nd and 98th percentile of the histogram voltage values of the signals. Apart from the well-known advantages of the normalization step in machine learning, this transformation also removes undesired peaks or noisy artifacts that may potentially distort the performance of the models.

### 4.4. Classification Models

#### 4.4.1. SimpleCNN

The first model we tested consisted of a SimpleCNN architecture that served as the baseline to analyze the detection problem. This network included 3 fully connected layers with 128 units, 64 units, and 1 unit, respectively. We used a rectified linear unit (ReLU) as the activation function and sigmoid output for binary classification.

#### 4.4.2. ATI-CNN

We tested a deep neural network model (ATI-CNN) used in ECG multi-class arrhythmia detection [6]. The idea was to employ a network with a good classification accuracy and with similar input data, i.e., ECG signals. This architecture implements a 13-layer fully CNN with a kernel size of 3 and increasing CNN (64, 128, 256, 256, and 256) kernels of size 3 with pooling layers. This first block eases the spatial fusion of the data from different signals. Then, two LSTM cells follow the convolutional block to achieve temporal data fusion, which allows the temporal flow information transmission between the cells. The cells decide to forget or remember part of the information from the feature vectors fed by the previous convolutional block.

#### 4.4.3. CRNN

The detailed network architecture implemented for the CRNN model is included in Table 3. The model takes as its starting point the AlexNet [50], with improvements to fit the input rectangular shape in terms of kernel, pooling, and stride sizes. To capture the temporal information of the signals, we added two GRU layers of 32 units each before the final output sigmoid layer. This approach is based on previous literature on audio tagging [51] and ECG detection problems [52]. 

The final architecture in Table 3 consists of 3 CNN blocks and 2 GRU layers. First, we added zero padding to the input signals and batch normalization. For the CNN blocks, rectangular 5 × 23 kernels were used with batch normalization and leaky ReLU activation function. Max-pooling was increased in each block, with dropout set at 30% to reduce overfitting. The GRU layers included 32 units each.

### 4.5. Implementation and Specifications

This study was developed in Python™ v3.8 in the 64-bit version using PyCharm 2021.1.2 (JetBrains, Prague, Czech Republic). The DL models were developed using Keras [29] with CUDA v11.0 and cudnn 8.0.4.30 (NVIDIA Corporation, Santa Clara, CA, USA). Data were processed using an Intel^®^ Core™ i9 CPU 3.10 GHz processor, 32 GB RAM, and Nvidia GeForce RTX 2070 Super GPU on Windows 10 64 bits. The models were trained with an Adam optimizer [53], with a grid search for learning rates of [10^−2^, 10^−3^, 10^−4^], binary cross-entropy as the loss function, and a batch size of 32.

### 4.6. Statistical Analysis and Performance Metrics

We present categorical values as absolute and relative percentages (frequencies), and normally distributed variables are summarized by the mean and standard deviation (SD). We tested the continuous variables using One-Way ANOVA, including Tukey honestly significant difference (HSD). The analysis was performed in Python (PyCharm 2020.1.3, JetBrains, Prague, Czech Republic) and JMP statistical software version 14.3.0 (SAS, Cary, NC, USA). In all tests, a *p*-value < 0.05 was considered statistically significant.

To compare the models that presented the best performance in the classification problem, we calculated their accuracy, precision, recall (sensitivity), specificity (selectivity), and *MCC* correlation coefficient, as follows:

(1) $$Accuracy=\frac{TP+TN}{TP+FP+FN+TN},$$

(2) $$Precision=\frac{TP}{TP+FP},$$

(3) $$Recall=\frac{TP}{TP+FN},$$

(4) $$Specificity=\frac{TN}{TN+FP},$$

(5) $$MCC=\frac{TP\cdot TN-FP\cdot FN}{\sqrt{\left(TP+FP\right)\cdot \left(TP+FN\right)\cdot \left(TN+FP\right)\cdot \left(TN+FN\right)}},$$

where *TP*, *FN*, *TN*, and *FP* stand for true positive, false negative, true negative, and false positive, respectively, and are the elements of the confusion matrix that summarize the prediction results of a model. *MCC* is the Matthews correlation coefficient that is commonly used in machine learning to measure the quality of a binary classification problem and provides a more reliable statistical rate using the values of the confusion matrix [54]. *MCC* provides a high score if all four confusion values are good, and it proportionally takes into account both positive and negative dataset elements.

## 5. Conclusions

In this study, we propose a novel detection algorithm for rotational activity detection based on CRNNs. The algorithm successfully detected the presence of rotors with an accuracy of 80.04% using bipolar EGMs and 68.40% with unipolar EGMs and was compared against two CNN models in the literature. The algorithm also outperformed the detection capabilities over unipolar signals when bipolar EGMs were employed, while improving the computational times compared to the aforementioned algorithm employed for labeling the data. The results obtained by employing DL methods in clinical detection problems open new research opportunities in cardiology.

## Figures and Tables

**Table 1:** Baseline characteristics of the patients.

Characteristics	All Patients	Training Set	Test Set	*p*-Value
N	75 (100.0)	43	5	-
Age (years)	60.7 ± 9.7	61.4 ± 9.5	60.0 ± 4.1	0.7509
Sex				
Men	56 (74.7)	37 (86.0)	3 (60.0)	0.1389
Women	19 (25.3)	6 (14.0)	2 (40.0)	0.1389
Atrial volume (cm^3^)	148.5 ± 39.4	158.6 ± 40.5	144.4 ± 37.7	0.4649
Diagnosis of AF (years)	3.1 ± 3.5	3.6 ± 2.5	1.8 ± 1.5	0.1594
Comorbidities				
BSA (m^2^)	2.0 ± 0.2	2.1 ± 0.2	2.1 ± 0.1	0.6083
CHA_2_DS_2_-VASc	1.8 ± 1.5	1.6 ± 1.4	2.0 ± 1.3	0.5403
COPD	4 (5.3)	4 (9.0)	0 (0.0)	0.4777
Diabetes mellitus	12 (16.0)	6 (14.0)	1 (20.0)	0.7188
Dyslipidemia	26 (34.7)	16 (37.0)	3 (60.0)	0.3222
Heart failure	13 (17.3)	8 (16.6)	1 (20.0)	0.9362
Hypertension	36 (48.04)	21 (49.0)	3 (60.0)	0.6384
Obstructive sleep apnea	16 (21.3)	10 (23.0)	0 (0.0)	0.2263
SHD	22 (29.3)	12 (28.0)	3 (60.0)	0.1416
Stroke	4 (5.33)	4 (9.0)	0 (0.0)	0.4777
Signal acquisitions (per patient)				
Number of acquisitions	37.2 ± 14.7	39.9 ± 13.6	35.0 ± 8.6	0.4471
Number of rotational events	51.2 ± 112.4	66.5 ± 111.9	50.4 ± 57.2	0.7568
Rotor cycle duration (ms)	166.8 ± 36.1	167.0 ± 36.4	164.4 ± 31.1	0.9073

Values in the table are n (%) or mean ± standard deviation. AF, atrial fibrillation; BSA, body surface area; COPD, chronic obstructive pulmonary disease; SHD, structural heart disease.

**Table 2:** Performance results.

DL Model	Input Data	Signal Length	Sampling Frequency	Validation Accuracy	Test Accuracy	Precision	Recall	Specificity	MCC
	(Type)	(ms)	(Hz)	(%)	(%)	(%)	(%)	(%)	
**SimpleCNN**	**uEGMs**	500	500	49.83	49.93	49.96	77.82	22.05	−0.002
			250	50.99	49.07	49.25	60.72	37.43	−0.019
			100	53.54	52.13	52.26	49.38	54.89	0.043
		2500	500	51.23	49.97	49.94	26.88	**73.05**	−0.001
			250	48.82	50.62	50.37	84.33	16.91	0.017
			100	50.20	51.24	51.15	55.00	47.48	0.025
	**bEGMs**	500	500	50.00	50.00	50.00	**100.00**	0.00	0.000
			250	50.00	50.01	50.00	**100.00**	0.01	0.008
			100	48.96	49.46	49.27	36.73	62.18	−0.011
		2500	500	50.00	50.00	50.00	**100.00**	0.00	0.000
			250	50.00	50.00	50.00	**100.00**	0.00	0.000
			100	50.00	50.05	50.03	**100.00**	0.10	0.022
	**uLATs**	500	500	**57.90**	**55.86**	**55.10**	63.32	48.39	**0.118**
			250	55.97	53.21	52.87	59.08	47.34	0.065
			100	53.61	52.51	52.45	53.64	51.38	0.050
		2500	500	51.07	50.77	51.14	34.67	66.88	0.016
			250	51.36	49.80	49.78	44.77	54.84	−0.004
			100	52.16	48.59	48.62	49.97	47.20	−0.028
**ATI-CNN**	**uEGMs**	500	500	64.00	58.56	70.63	29.32	87.80	0.211
			250	58.82	56.83	73.20	21.54	92.11	0.193
			100	62.00	58.81	66.80	35.05	82.57	0.200
		2500	500	63.37	59.28	68.18	34.80	83.76	0.213
			250	59.85	58.83	71.11	29.73	87.92	0.217
			100	54.33	55.67	66.41	22.95	88.39	0.150
	**bEGMs**	500	500	62.69	63.30	71.97	43.56	83.04	0.289
			250	67.60	58.75	68.16	32.86	84.65	0.205
			100	67.42	62.00	69.78	42.33	81.67	0.261
		2500	500	64.01	59.85	63.46	46.44	73.26	0.204
			250	65.49	63.05	65.09	56.31	69.80	0.263
			100	65.74	58.96	57.69	67.21	50.70	0.182
	**uLATs**	500	500	64.14	63.29	69.56	47.25	79.33	0.281
			250	70.56	65.36	68.63	56.57	74.14	0.312
			100	**76.44**	**70.03**	69.48	71.44	68.62	**0.401**
		2500	500	-	-	-	-	-	- ^1^
			250	62.39	54.23	**75.75**	12.43	**96.02**	0.154
			100	70.89	65.46	59.85	**93.95**	36.97	0.376
**CRNN**	**uEGMs**	500	500	71.76	68.40	**77.91**	51.36	85.44	0.400
			250	65.76	63.12	77.17	37.25	88.98	0.310
			100	56.25	64.81	70.93	50.18	79.44	0.310
		2500	500	63.05	60.55	46.73	64.73	58.27	0.220
			250	71.40	63.66	70.90	46.34	80.98	0.290
			100	64.86	61.71	58.20	83.11	40.31	0.260
	**bEGMs**	500	500	78.39	72.52	67.12	89.80	50.24	0.410
			250	72.57	59.88	76.89	28.24	91.51	0.260
			100	73.18	65.48	67.85	58.82	72.13	0.310
		2500	500	**80.93**	**80.04**	74.14	92.27	67.82	**0.680**
			250	79.23	63.96	63.28	66.50	61.42	0.280
			100	74.64	60.33	63.47	48.70	71.97	0.210
	**uLATs**	500	500	74.28	68.72	67.09	73.46	63.98	0.376
			250	69.60	61.87	57.30	93.21	30.54	0.305
			100	73.15	64.61	60.96	81.27	47.95	0.310
		2500	500	50.48	49.84	30.77	0.26	**99.41**	−0.025
			250	67.86	56.94	53.87	**96.68**	17.20	0.229
			100	70.71	60.23	71.01	34.57	85.89	0.238

^1^ Training on the GPU could not be achieved due to a lack of memory. The best metrics for each model are highlighted with different background colors. DL, deep learning; MCC, Matthews correlation coefficient; CNN, convolutional neural network; ATI-CNN, attention-based time-incremental convolutional neural network; CRNN, convolutional recurrent neural network; uEGMs, unipolar electrograms; bEGMs, bipolar electrograms; uLATs, unipolar local activation times.

**Table 3:** CRNN model for rotational activity detection.

Layer	Kernel Size (H, W, D)	Stride (H, W)	Activations ^1^	
			Unipolar EGMs, LATs	Bipolar EGMs
Input	-	-	1250 × 20 × 1	1250 × 15 × 1
Zero Padding 2D 1	(37, 0, 0)		1324 × 20 × 1	1324 × 15 × 1
Batch Normalization 1	-	-		
Dropout 1	-	-	1324 × 20 × 1	1324 × 15 × 1
Conv2D 1	(5, 23, 32)	(1, 1)	1324 × 20 × 32	1324 × 15 × 32
Batch Normalization 2	-	-	1324 × 20 × 32	1324 × 15 × 32
LeakyReLU 1	-	-	1324 × 20 × 32	1324 × 15 × 32
Max Pooling 2D 1	(2, 2, 32)	(2, 1)	662 × 19 × 32	662 × 14 × 32
Dropout 2	-	-	662 × 19 × 32	662 × 14 × 32
Conv2D 2	(5, 23, 64)	(1, 1)	662 × 19 × 64	662 × 14 × 64
Batch Normalization 3	-	-	662 × 19 × 64	662 × 14 × 64
LeakyReLU 2	-	-	662 × 19 × 64	662 × 14 × 64
Max Pooling 2D 3	(3, 3, 64)	(3, 3)	220 × 6 × 64	220 × 4 × 64
Dropout 3	-	-	220 × 6 × 64	220 × 4 × 64
Conv2D 3	(5, 23, 64)	(1, 1)	220 × 6 × 64	220 × 4 × 64
Batch Normalization 4	-	-	220 × 6 × 64	220 × 4 × 64
Leaky ReLU 3	-	-	220 × 6 × 64	220 × 4 × 64
Max Pooling 2D 3	(4, 4, 64)	(4, 4)	55 × 1 × 64	55 × 1 × 64
Dropout 4	-	-	55 × 1 × 64	55 × 1 × 64
Reshape 1	-	-	55 × 64	55 × 64
GRU 1	32 units	-	55 × 32	55 × 32
GRU 2	32 units	-	32	32
Dropout 5	-	-	32	32
Dense	-	-	1	1

^1^ The activations in the table are for 2500 ms signals at sampling frequency 500 Hz. The different activations for the network input size variations can be extrapolated from the kernel and stride columns. CRNN, convolutional recurrent neural network; H, height; W, width; D, depth; EGMs, electrograms; LATs, local activation times; GRU, gated recurrent unit; ReLU, rectified linear unit.

## Data Availability

The data of this study are available from the corresponding author on request.
